# CAR T Cells Targeting the Tumor MUC1 Glycoprotein Reduce Triple-Negative Breast Cancer Growth

**DOI:** 10.3389/fimmu.2019.01149

**Published:** 2019-05-24

**Authors:** Ru Zhou, Mahboubeh Yazdanifar, Lopamudra Das Roy, Lynsey M. Whilding, Artemis Gavrill, John Maher, Pinku Mukherjee

**Affiliations:** ^1^Department of Biological Sciences, University of North Carolina at Charlotte, Charlotte, NC, United States; ^2^School of Cancer and Pharmaceutical Sciences, King's College London, Guy's Hospital Campus, London, United Kingdom

**Keywords:** triple-negative breast cancer, immunotherapy, MUC28z CAR T cells, MUC1, TAB004

## Abstract

Antibody-derived chimeric antigen receptor (CAR) T cell therapy has achieved gratifying breakthrough in hematologic malignancies but has shown limited success in solid tumor immunotherapy. Monoclonal antibody, TAB004, specifically recognizes the aberrantly glycosylated tumor form of MUC1 (tMUC1) in all subtypes of breast cancer including 95% of triple-negative breast cancer (TNBC) while sparing recognition of normal tissue MUC1. We transduced human T cells with MUC28z, a chimeric antigen receptor comprising of the scFv of TAB004 coupled to CD28 and CD3ζ. MUC28z was well-expressed on the surface of engineered activated human T cells. MUC28z CAR T cells demonstrated significant target-specific cytotoxicity against a panel of human TNBC cells. Upon recognition of tMUC1 on TNBC cells, MUC28z CAR T cells increased production of Granzyme B, IFN-γ and other Th1 type cytokines and chemokines. A single dose of MUC28z CAR T cells significantly reduced TNBC tumor growth in a xenograft model. Thus, MUC28z CAR T cells have high therapeutic potential against tMUC1-positive TNBC tumors with minimal damage to normal breast epithelial cells.

## Introduction

Triple-negative breast cancer (TNBC) is characterized for being negative in the expression of human epidermal growth factor receptor 2 (HER2), estrogen receptor (ER), and progesterone receptor (PR), and accounts for ~15% of invasive breast cancers (1, 2). There are limited treatment options for patients with TNBC. Patients cannot benefit from HER2-targeted therapy, and have worse outcome after chemotherapy compared to breast cancer patients with other subtypes (3). Thus, development of newer therapies, such as immunotherapy, for TNBC patients is warranted.

Chimeric antigen receptors (CARs) are synthetic receptors that when engineered into T cells, they can redirect T cells to recognize a tumor-specific antigen, get activation to become cytolytic, and eventually lyse the tumor cells (4, 5). The CAR T cell approach combines an extracellular single chain variable fragment (scFv) from an antibody that can recognize a tumor surface antigen, a transmembrane domain, and a T cell intracellular signaling domain into a fusion molecule for tumor cell lysis (6). Despite its success in treating hematologic malignancies (2, 5, 7), CAR T cell immunotherapy remains a challenge for solid tumors due to lack of specific targetable cell surface antigens. An ideal tumor target antigen should be highly restricted to tumor cells, and only minimally detected in normal tissues. Mucin1 glycoprotein (MUC1) has been suggested as one of the favorable targets for engineering CAR T cells (8–10).

MUC1 occurs naturally as a heavily glycosylated transmembrane mucin protein that is normally expressed on all glandular epithelial cells that form our major organs including breast. The large extracellular domain of MUC1 contains variable number tandem repeats (VNTR) region. The VNTR region is consisted of 25–125 repeats of a 20 amino acid sequence, mainly comprised of proline, serine, and threonine residues. The enriched serine and threonine residues serve as a scaffold for the attachment of O-glycans, allowing them to undergo extensive O-linked glycosylation (11–13). Modifications in the O-glycosylation generates a classical tumor antigen known as the Tn antigen. The addition of a sialyl-residue is the last step in O-glycosylation (14). A further glycosylation of core 1 glycans forms the core 2 elongated glycans. In normal healthy cells, MUC1 is mainly covered with extensively elongated and branched core 2 glycans. As cells transform to a malignant phenotype, expression of MUC1 increases several fold, and glycosylation on tumor synthesized MUC1 is aberrant by reduction of core 2 glycans and a predominance of core 1 glycans (15). This process leads to the exposure of protein epitopes that are normally not accessible in normal heavily glycosylated MUC1 (16). Little is known regarding the specifics of tumor-associated MUC1 (tMUC1) epitope, but thus far, tumor MUC1 targeting is mostly restricted to the VNTR region.

MUC1 is over-expressed and aberrantly glycosylated in more than 90% of breast cancer cases. This tumor-associated MUC1 is a marker of an aggressive phenotype (17), and serves as a tumor neoantigen for targeted immunotherapy. We have developed a novel monoclonal antibody TAB004 (Patents *US 8518405* B2 &* US 9090698* B2) (18, 19) that is highly specific for the tMUC1 and does not recognize normal epithelia (18, 19). TAB004 recognizes the altered glycosylated epitope within the MUC1 tandem repeat sequence, and its binding epitope is the sequence STAPPVHNV (18). We recently published that 95% of all malignant tissues (including TNBC) are targeted by TAB004 indicating their expression of tMUC1. From a panel of 13 human TNBC cell lines, 11 showed higher frequency of tMUC1 expression compared to normal breast epithelial cells (19). When injected into human TNBC (HCC70) tumor-bearing mice or the PyVMT.MUC1 transgenic mice (that develop spontaneous mammary gland tumors), TAB004 accumulated only in the tumor, but not in any other organs (19, 20). Thus, TAB004 detects tMUC1 in a highly specific manner, sparing recognition of normal tissues. Therefore, we utilized TAB004 to engineer the MUC28z fusion molecule for generating the CAR T cells. MUC28z comprised of the scFv sequence derived from TAB004, fused to CD28 and CD3ζ T cell intracellular signaling molecule.

In this study, we generated the MUC28z CAR T cells and performed phenotypic and functional analysis of these T cells. We found that MUC28z CAR T cells had high tumor antigen specificity and strong tumor cytolytic efficacy for TNBC, both *in vitro* and *in vivo*.

## Materials and Methods

### Ethical Approval

All applicable international, national, and/or institutional guidelines for the care and use of animals were followed. All procedures performed in studies involving animals were in accordance with the ethical standards of the institution and approved by the Institutional Animal Care and Use Committee of the University of North Carolina at Charlotte.

### Cell Lines

The TNBC cell lines for this study were purchased from American Type Culture Collection (ATCC, Manassas, VA 20110, USA) and selected from ATCC Breast Cancer Cell Panel (ATCC 30-4500K). Cell lines were cultured as instructed. The retroviral vector packaging line GP2-293 cell line was cultured in DMEM supplemented with 10% fetal bovine serum, 1% penicillin/streptomycin and 1% glutamax (all obtained from ThermoFisher Scientific, Waltham, MA). The normal breast epithelial cells AG11132 and AG11134 were purchased from Coriell Institute for Medical Research (Camden, New Jersey). Cells were cultured in MEGM BulletKit as instructed (Lonza, Basel, Switzerland).

### Construction of Chimeric Antigen Receptor and Retroviral Vector Production

The second-generation of tMUC1-specific CAR construct (MUC28z) was synthesized by subcloning the scFv from the TAB004 (19) into the SFG-based retroviral backbone plasmid encoding the transmembrane and intracellular domains of CD28 and the intracellular domain of CD3ζ [kindly provided by Dr. John Maher group, details as described in Whilding et al. (21)]. A Myc epitope-tagged framework was engineered to enable the tracking of MUC28z expression.

To produce the MUC28z-encoding retroviral supernatants, GP2-293 cells were transfected with the SFG-retroviral plasmid via SuperFect (Qiagen, Germantown, MD) to produce virus with VSV envelop. Virus supernatants were collected 48 and 72 h after transfection.

### T Cell Transduction

CAR T cells engineered from mixed populations of CD8^+^ and CD4^+^ T cells have demonstrated better outcomes, both in mouse models (5, 22, 23) and in certain clinical studies (5, 7, 24, 25). Therefore, the unfractionated peripheral blood mononuclear cells (PBMCs) from healthy donors were applied in our studies (purchased from STEMCELL Technologies, Cambridge, MA). Human PBMCs were activated with anti-CD3/CD28 beads (ThermoFisher Scientific, Waltham, MA). On day 3, activated T cells were transduced with the retroviral supernatants and spinoculated for 1 h at 1,000 g on plates coated with Retronectin (Takara, Mountain View, CA). Transduced T cells were maintained in IL-2 (10 ng/ml, PeproTech, Rocky Hill, NJ). On day 6, the anti-CD3/CD28 beads were removed from cultures. On day 7, cells were supplemented with IL-7 and IL-15 (10 ng/ml each, PeproTech). The transduction efficiency and cell phenotype were monitored by flow cytometry analysis. Activated but non-retroviral transduced cells were included as mock T controls.

### T Cell Functional Assays

The antigen-specific tumor cell lysis by MUC28z CAR T cells was determined by MTT assay. Briefly, a panel of TNBC cell lines were plated in 96-well plates overnight. The next day, culture media from the plate was aspirated and MUC28z CAR T cells were added at the indicated E:T ratio. At 24, 48, and 72 h after co-culture, T cells were removed and replaced with fresh media containing MTT (500 μg/ml, Sigma) for 3 h. After the uptake of MTT, supernatants from wells were discarded and 150 μl of DMSO was added to wells to dissolve the formazan crystals. The OD value was read at 540 nm. We used the mock T cell lysis data for calculating % lysis. The calculation formula was: (OD of co-culture with mock T – OD of co-culture with CAR T)/OD of co-culture with mock T × 100.

The antigen-specific cytokine release was performed by co-culturing MUC28z CAR T cells with HCC70 cells at E:T of 2:1 ratio. After 24 h of co-culture, supernatants were collected. The IFN-γ concentration was determined using Duoset Human Cytokine Detection Kit in accordance with the manufacturer's instructions (R&D Systems; Minneapolis, MN, USA). The panel of cytokines was detected using Proteome Profiler Human Cytokine Array Kit, Panel A (R&D Systems) as instructed. The array data were quantitated by ImageJ software.

### Flow Cytometry

The transduction efficiency of human MUC28z was evaluated via Myc-tag-FITC staining (Cell Signaling Technologies, Danvers, MA). T cell phenotypes were determined by staining for CD3-FITC, CD4-PE/Cy7, CD8-eF450, CD11c-APC (BD Biosciences, San Jose, CA), and for CD25-PE (eBioscience-ThermoFisher Scientific). Antibodies for PD1-APC (eBioJ105), IFN-γ-APC, and Granzyme B-PE were also obtained from eBioscience. The human tMUC1 expression on TNBC tumor cells was assessed by TAB004 staining (provided by OncoTab Inc., Charlotte, NC) conjugated with APC/Cy5.5 (Abcam; Cambridge, MA). Dead cells were excluded by 7-AAD staining (BD Biosciences). Data were acquired on BD LSRFortessa flow cytometer (BD Biosciences), and analyzed with FlowJo software (version 8.8.7, Tree Star Inc).

### Orthotopic Breast Cancer Animal Model and Analysis

The orthotopic breast cancer mouse model was performed using female NSG mice (NOD.Cg-Prkdc^scid^ Il2rg^tm1Wjl^/SzJ, 7–8 weeks of age, The Jackson Laboratory, Maine, USA). Mice were injected at one mammary fat pad with 5 × 10^6^ HCC70 cells resuspended in 100 ul of PBS: growth factor-reduced matrigel mixture (1:1 ratio; matrigel purchased from BD Biosciences). When tumor size reached ≥5 mm in diameter, mice were randomly assigned into experimental groups. The HCC70 tumor-bearing mice were adoptively transferred with 1 × 10^7^ transduced MUC28z CAR T cells (in 200 μl of PBS) via intravenous injection. Control mice received 200 ul of PBS only. Tumor growth was monitored by caliper measurement, twice a week. The tumor volume was calculated according to the formula: tumor volume (mm^3^) = [length in mm × (width in mm)^2^]/2. We used 7 mice per group for the 57-day study. We used 5 mice in the vehicle group and 4 mice in the MUC28z T treatment group for the 81-day study.

Tumor wet weight were measured at the endpoint. Tumor tissues were individually processed for single cell suspensions. A portion of cells was stained for CD45, CD4, CD8, CD25, or PD1, and immediately analyzed by flow cytometry to evaluate the MUC28z CAR T cell activation status *ex vivo*.

### IHC Staining

Tissues were fixed in 10% neutral-buffered formalin. Paraffin-embedded blocks were prepared by the Histology Core at the Carolina Medical Center and 4-μm-thick sections were cut for staining. IHC was performed as described previously (19, 26). Basically, the tumor sections were deparaffinized in xylene, rehydrated in a series of ethanol (100, 95, and 70%) followed by tap water and PBS, and then subjected to antigen retrieval in 99°C water bath for 40 min. The activity of endogenous peroxidases was blocked by 2% hydrogen peroxide for 15 min. The slides were washed twice in PBS and blocked with 50% FBS for 1 h at room temperature and then incubated with horseradish peroxidase-conjugated TAB004 (1:375 dilution) overnight at 4°C. The tissues were washed twice in PBS and the substrate 3,3′-diaminobenzidine (Vector Laboratories, Burlington, CA) was added for 5 min, followed by counterstaining with Mayer's hematoxylin solution. The tissues were then dehydrated in a series of ethanol, immersed into xylene and mounted using Permount (Fisher Scientific). Representative images were taken at × 100 magnification.

### Statistical Analysis

Data were analyzed using Prism (version 7.0; GraphPad Software) and results were presented as mean ± SD or mean ± SEM where indicated. Data were representative of two or more independent experiments. The statistical analysis was performed with Prism software and significance was determined by unpaired student *t*-test or two-way ANOVA (^*^p < 0.05, ^**^p < 0.01, ^***^p < 0.001). The number of mice chosen for *in vivo* treatment was based on power analysis for comparing the main effect of treatment, with *p* < 0.05, and Power level = 0.8.

## Results

### Enrichment of MUC28z CAR T Cells

**Figure 1:**
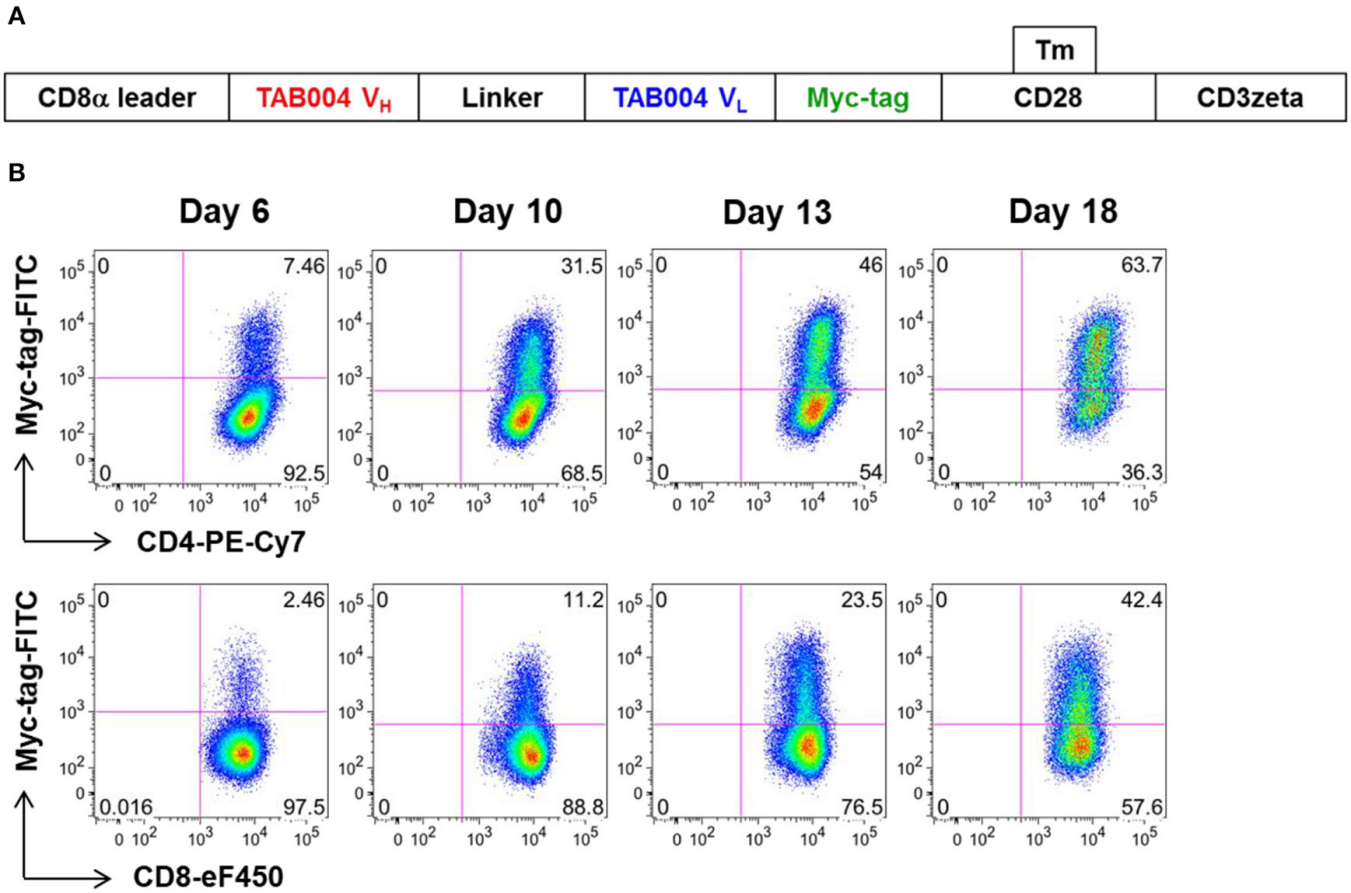
Increased MUC28z positivity on activated human PBMC. **(A)** Schematic diagram of the engineered receptor MUC28z. **(B)** MUC28z CAR expression in activated human T cells after retrovirus transduction, as determined by flow cytometry analysis of Myc-tag expression. Cells were gated for CD4 or CD8, and then analyzed for Myc-tag expression. Dead cells were excluded by 7-AAD staining.

We constructed a human CAR (MUC28z) that incorporated the scFv motif derived from TAB004, and the CD28/CD3ζ signaling domains. Figure 1A showed the schematic structure of the MUC28z CAR. After retrovirus transduction in activated human PBMCs, MUC28z CAR expression on activated T cells and MUC28z CAR T cell enrichment were monitored by Myc-tag staining and analyzed by flow cytometry. By day 18 after transduction, there were ~30–40% of Myc-tag^+^ cells within CD8^+^T cells, and 50–60% of Myc-tag^+^ cells within CD4^+^T cells (Figure 1B). In the following studies, we used the entire population of transduced T cells as MUC28z CAR T cells without further purification. MUC28z CAR T cells and mock (untransduced) T cells proliferated at the same rate *in vitro* until day 7, thereafter, MUC28z CAR T cells exceeded the expansion rate over mock T cells (Supplementary Figure 1).

### MUC28z CAR T Cells Mediate tMUC1-dependent TNBC Tumor Cell Lysis *in vitro*

**Figure 2:**
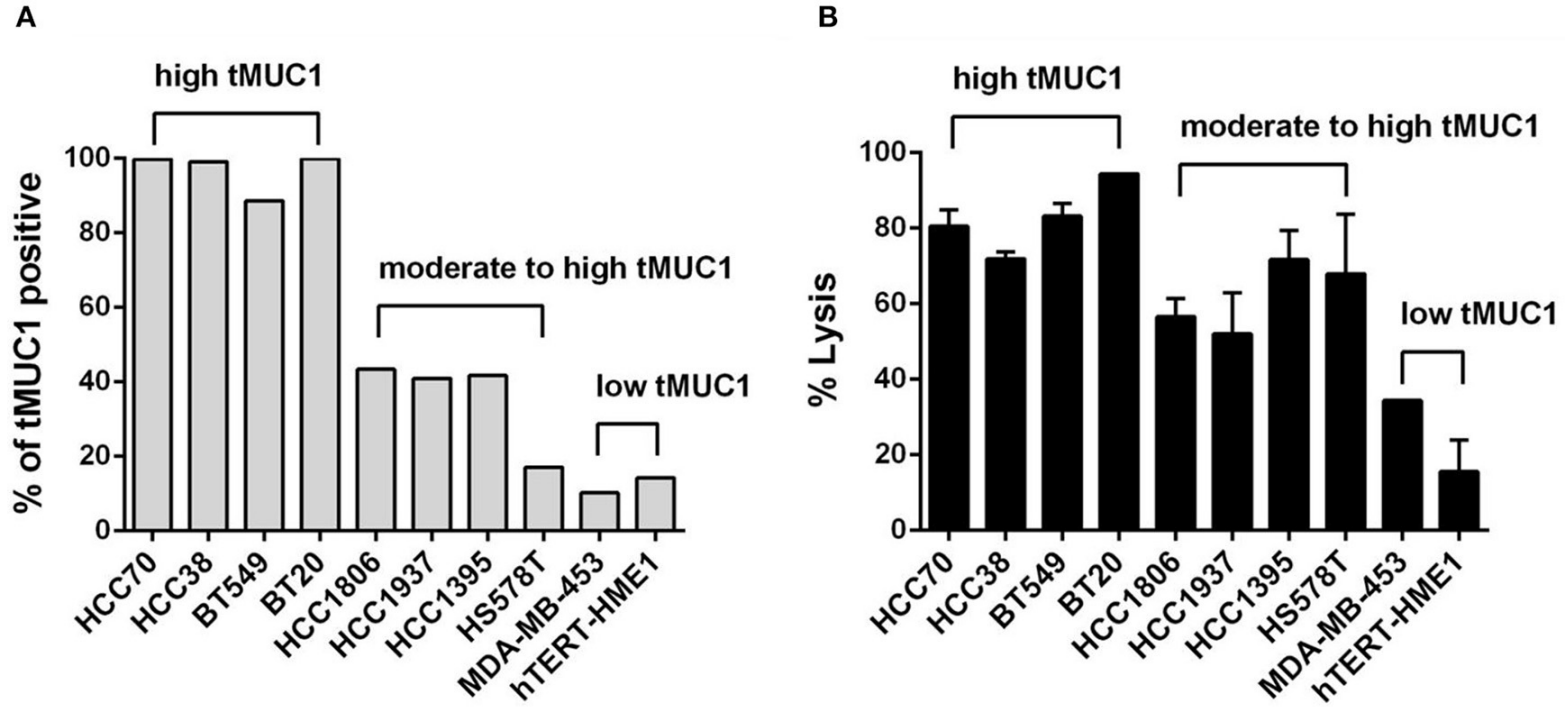
The MUC28z CAR T cells lyse TNBC tumor cells *in vitro* in an antigen-dependent manner. **(A)** Percentage of cells expressing tMUC1, determined by TAB004-APC/Cy5.5 staining and flow cytometry. A panel of nine TNBC cell lines and one “normal” mammary epithelial cell line hTERT-HME1 is shown. **(B)** Percentage of TNBC tumor cell lysis by MUC28z CAR T cells. Cells were co-cultured at E:T ratio of 5:1 for 3 days. The lysis of tumor cells was determined by MTT assay. Data are presented as the mean ± SEM. The relationship between tMUC1 positivity in TNBCs and tumor lysis by MUC28z CAR T cells was analyzed by a non-parametric Spearman correlation, with *r* = 0.8909 which was highly significant (*P* = 0.0011) and indicated a positive association between tMUC1 level and tumor lysis.

We first assessed the level of tMUC1 on the cell surface of a panel of TNBC cell lines by flow cytometry. Data are presented as percentage of cells that express tMUC1 (Figure 2A). We grouped cells based on tMUC1 frequency, from high, moderate, to low. The mean fluorescence intensity (MFI) of tMUC1 positive cells was presented as Supplementary Figure 2A. Next, we assessed tumor cell lysis efficacy of MUC28z CAR T cells. TNBC cells were co-cultured with MUC28z CAR T cells at a E:T ratio of 5:1 for 72 h. Based on the tMUC1 frequency, there was significant lysis of TNBC cell lines *in vitro* by the MUC28z CAR T cells (Figure 2B). One exception was the HS578T cell line that had very low levels of tMUC1 but had significant lysis by MUC28z CAR T cells. Currently, we are not sure why that is except to suggest that these cells are intrinsically highly sensitive to immune cell killing. All lysis data presented for all TNBC cell lines was normalized to its own mock T cell lysis. Using Spearman correlation analysis, the efficacy of MUC28z CAR T cells in TNBC cytolysis closely corresponded with the frequency of tMUC1 expressed by TNBC cells, with correlation *r* = 0.8909 (Spearman non-parametric analysis), indicating a tMUC1-dependent tumor cell killing. hTERT-HME1 was used as a “normal” breast epithelial cell line that expressed minimal levels of tMUC1 and consequently showed minimal lysis by MUC28z CAR T cells.

We also performed the tumor lysis at a E:T ratio of 2:1 for 24 and 48 h (shown in Supplementary Figures 2B,C). Data were consistent with the results observed in Figure 2B, with the exception of HCC1806 cell line which seemed to require 5:1 of E:T ratio and longer (72 h) incubation with CAR T cells as seen in Figure 2B. We obtained AG11132 and AG11134, two epithelial cells from breast organoid which were sampled from apparently healthy individuals. Those two cells barely express tMUC1 (Supplementary Figure 2D, left panel). When co-cultured with mock T cells or MUC28z CAR T cells at a E:T ratio of 2:1, MUC28z CAR T cells showed no significant lysis against AG11132 and AG11134 (Supplementary Figure 2D, right panel), suggesting limited off-target lysis in normal breast epithelial tissues by our MUC28z CAR T cells.

### Cell Surface Markers on MUC28z CAR T Cells Suggest Highly Activation Cells

First, we determined that >92% of MUC28z CAR and mock transduced PBMCs were indeed T cells. There were no CD14^+^, CD16^+^, CD19^+^, or CD56^+^ cells in the population that was used for *in vitro* or *in vivo* cytotoxic activity (data not shown). Since both CD4^+^ and CD8^+^ MUC28z CAR T cells displayed similar phenotypical changes, we only show data from CD8^+^ population here.

**Figure 3:**
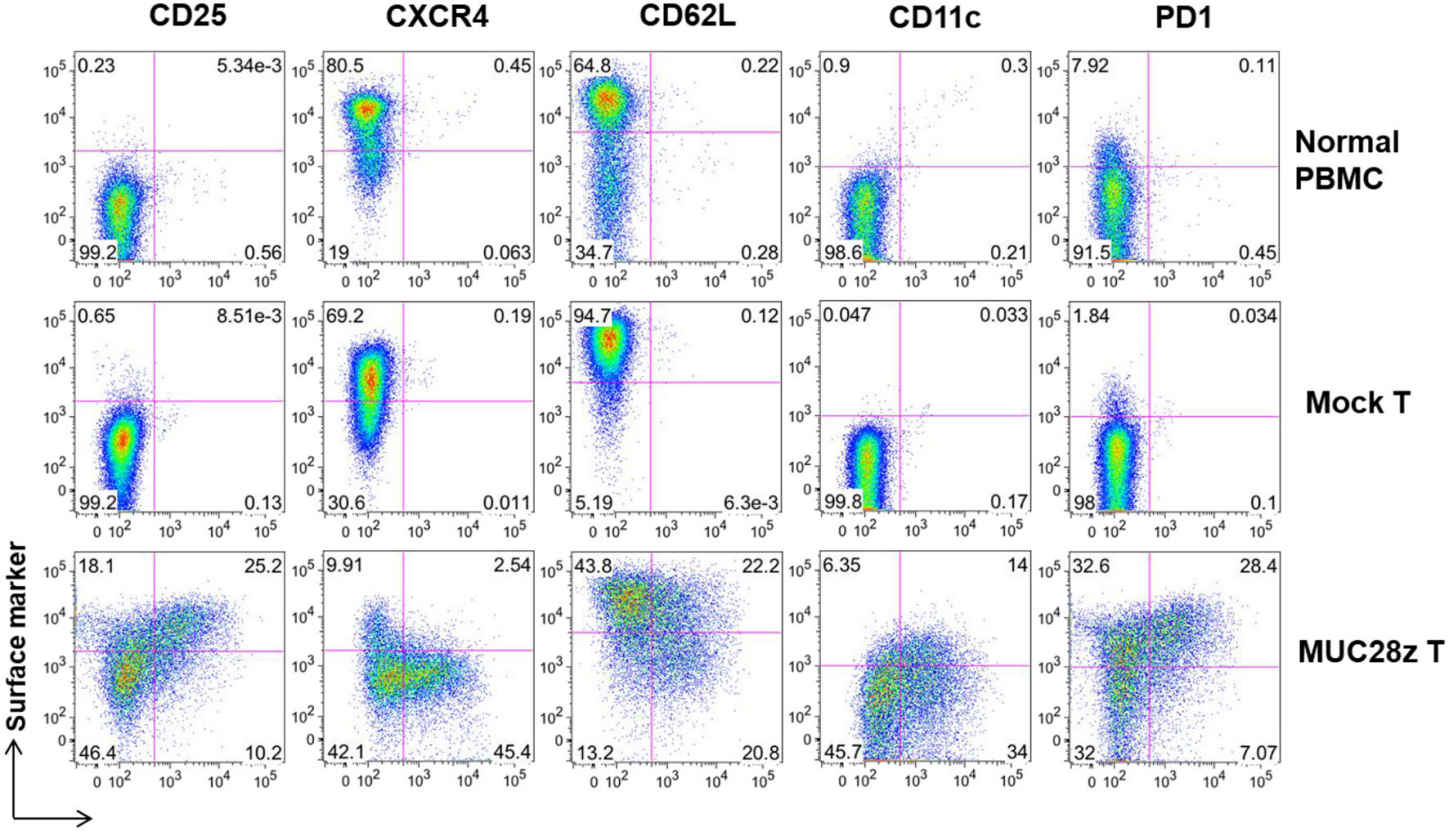
MUC28z CAR T cells show significant difference in cell surface marker expressions *in vitro*. The expression of the indicated surface markers on MUC28z CAR T cells was determined by flow cytometry on day 14 after retrovirus transduction. Cells were gated on CD8^+^ T cells. The PBMCs from normal healthy donor were cultured overnight without stimulation and served as normal PBMC control.

Next, a panel of cell surface markers that represents activated phenotype of T cells were assessed by flow cytometry. Compared to controls (mock and normal PBMCs), there was a significant increase in CD25, CD11c, and PD1 expression on the total CD8^+^Myc-tag^+^ MUC28z CAR T cells suggesting highly activated phenotype (Figure 3). Furthermore, data shows dramatically reduced expression of CXCR4 on CD8^+^MUC28z CAR T cells compared to controls, suggesting their post-activation status corresponding with the up-regulation of CD25. It has been demonstrated that CXCR4 expression is down-regulated on human CD8^+^ T cells during peripheral differentiation (27). The lower level of CD62L on MUC28z CAR T cells suggested that there were probably more effector cell populations. We also found that a significant portion of CD8^+^Myc-tag^+^ MUC28z CAR T cells co-expressed CD11c. CD11c^+^CD8^+^ T cells are a subset of activated, antigen-specific cytotoxic T cells (28). There was high expression of PD1 on CD8^+^ MUC28z CAR T cell surface suggesting the possible cell exhaustion.

### MUC28z CAR T Cells Are Highly Activated When Co-cultured With tMUC1-positive TNBC Cells: Increased Release of Cytokines, Chemokines, and Granzyme B

**Figure 4:**
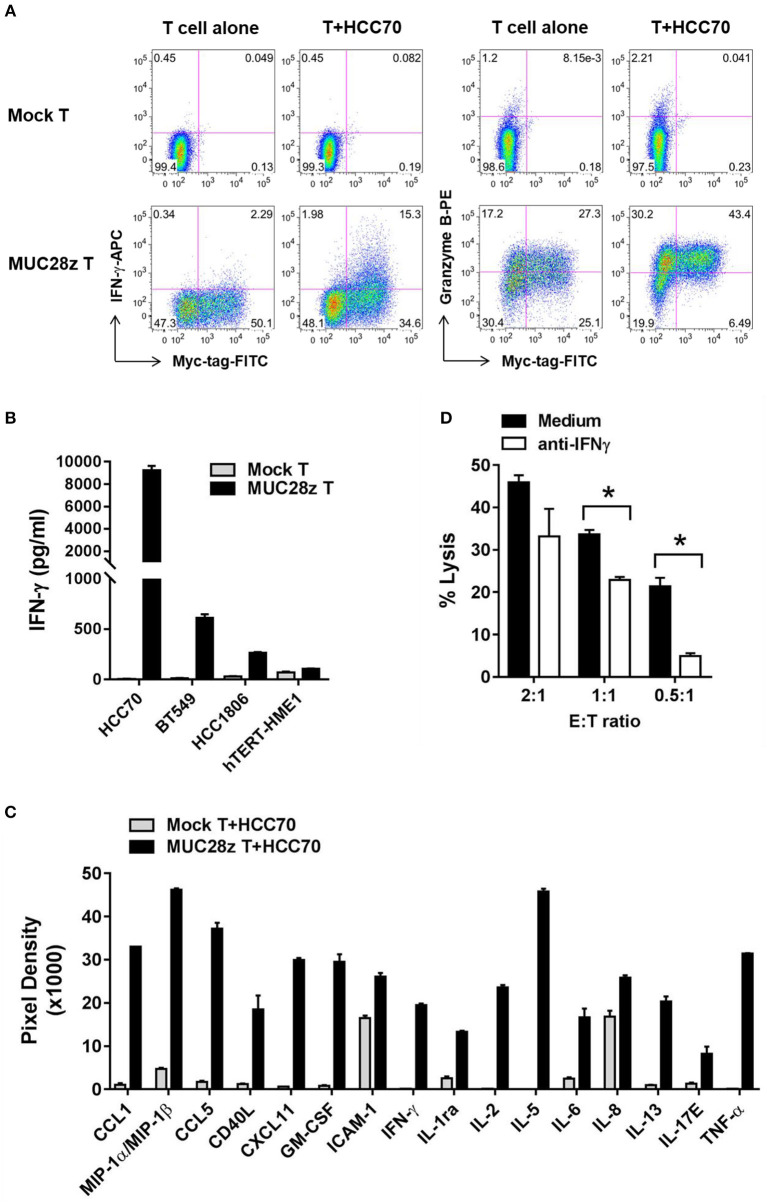
MUC28z CAR T cells release antigen-specific cytokines when co-culture with tMUC1^high^ TNBCs. **(A)** CD8^+^ MUC28z CAR T cells produced IFN-γ and increased Granzyme B in response to tMUC1^high^ HCC70 cells *in vitro*. MUC28z CAR T cells were co-cultured with HCC70 tumor cells for 24 h, and then cells were stained intracellularly for IFN-γ and Granzyme B in addition to Myc-tag. Cells were gated on CD8^+^ T cells, followed by flow cytometry analysis. **(B)** MUC28z CAR T cells produced IFN-γ in response to tMUC1-expressing TNBC *in vitro*. T cells were co-cultured with the selected tumor cell lines (E:T = 2:1) for 24 h, and then the culture supernatants were assayed for IFN-γ by ELISA. Data are presented as mean ± SD of replicates. The baseline IFN-γ releases in the absence of TNBC stimulation are: mock T = 2.6 ± 0.9 pg/ml; MUC28z CAR T = 18.7 ± 0.1 pg/ml. **(C)** MUC28z CAR T cells released a large panel of cytokines after tMUC1-specific cell activation. T cells were co-cultured with HCC70 cells (E:T = 2:1) for 24 h, and then the culture supernatants were assayed for cytokine concentration by human cytokine array. Data are presented as mean ± SD of replicates. **(D)** The lysis of HCC70 cells by MUC28z CAR T cells was partially reversed by IFN-γ neutralization. HCC70 cells were co-cultured with MUC28z CAR T cells for 24 h in the absence or presence of IFN-γ neutralizing antibody. The lysis of HCC70 cells was determined by MTT assay. Data are presented as the mean ± SEM. *p < 0.05 (student *t*-test).

In this experiment, MUC28z CAR T cells or mock T cells were co-cultured with TNBC cell lines for 24 h. Thereafter, supernatants were collected and tested for cytokine release. We first tested the release of IFN-γ and Granzyme B in response to co-culture with HCC70 (high tMUC1 expressing) cells. Mock T cells remained “quiescent” with minimal release of IFN-γ and Granzyme B because there was no stimulatory signal between the tumor cells and mock T cells (Figure 4A, top). In contrast, after co-culture with HCC70 cells for 24 h, MUC28z CAR T cells showed significant increases in IFN-γ and Granzyme B production in the CD8^+^Myc-tag^+^ MUC28z CAR T cells (Figure 4A, bottom). Next, we measured the production of IFN-γ from MUC28z CAR T cells in the supernatants post co-culture with TNBC cell lines including HCC70 (100% of cells positive for tMUC1), BT549 (86% cells positive for tMUC1), and HCC1806 (40% cells positive for tMUC1). In addition, MUC28z CAR T cells were co-incubated with normal breast epithelial cell line, hTERT-HME1 which expresses minimal levels of tMUC1. Data in Figure 4B clearly shows that the IFN-γ production by MUC28z CAR T cells required tMUC1 antigen expression on TNBC cells. Importantly, there was minimal production of IFN-γ when MUC28z CAR T cells were co-cultured with “normal” breast epithelial cell line (hTERT-HME1). Yet again, mock T cells showed no release of IFN-γ when co-cultured with the same TNBC cell lines. Similar changes were observed when MUC28z CAR T cells were co-cultured with other TNBC cell lines (Supplementary Figure 3). Taken together, Figures 4A,B demonstrated that the upregulation of IFN-γ and Granzyme B release was clearly dependent upon the tMUC1 antigen binding to the scFv of TAB004 on the cell surface of MUC28z CAR T cells. It is recognized that although BT549 and HCC70 expressed very similar levels of surface tMUC1 and lysis by MUC28z CAR T cells, the levels of IFN-γ secretion were significantly different between the two cell lines, with HCC70 secreted very high levels. We do not have any explanation for this difference at this time but needless to suggest that intrinsic genetic variability between the cell lines may account for the differences.

Using a human cytokine array, we found that in comparison to mock T cells, there was a dramatic increase in release of several cytokines and chemokines by MUC28z CAR T cells (Figure 4C) when co-cultured with HCC70 cells for 24 h. These data confirmed that MUC28z CAR T cells recognized the tMUC1 antigen and became highly activated and lytic against MUC1-positive TNBCs as compared to activated mock T cells.

To further determine if the cytolytic activity of MUC28z CAR T cells was IFN-γ-dependent, we neutralized IFN-γ with anti-IFN-γ antibody during co-culture, and found that IFN-γ neutralization significantly compromised the tumor lysis ability of MUC28z CAR T cells at E:T ratio of 0.5:1 and 1:1 and trended to compromise lysis at 2:1 of E:T ratio (Figure 4D) suggesting that the lytic potential of the effector cells is partially dependent upon IFN-γ. The target tumor cell line for Figure 4D was HCC70 cells.

### MUC28z CAR T Cells Control HCC70 Tumor Growth *in vivo*

**Figure 5:**
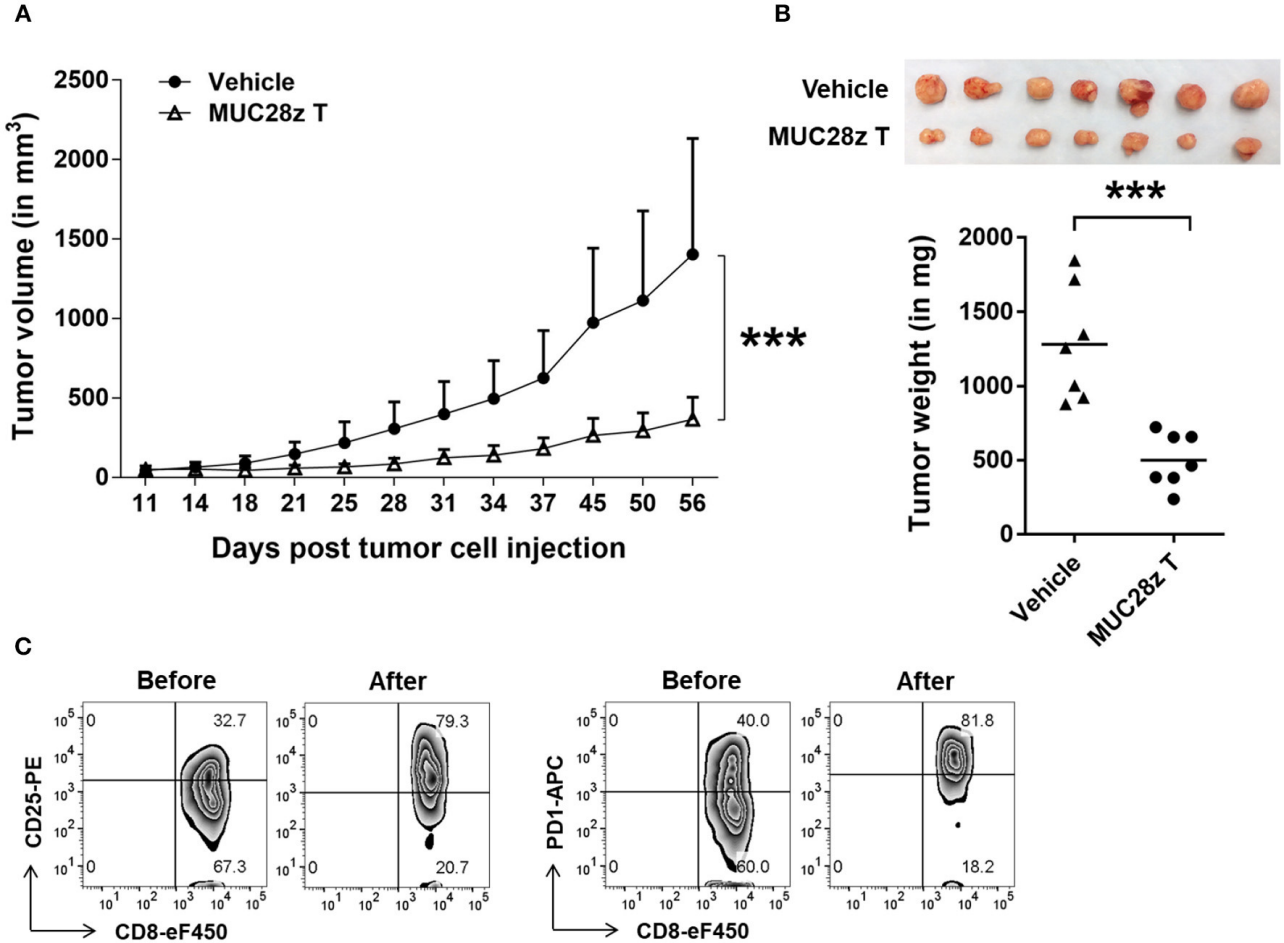
MUC28z CAR T cells reduce HCC70 tumor burden *in vivo*. **(A)** HCC70 tumor growth reduction by a single injection of MUC28z CAR T cells *in vivo*. HCC70 cells were orthotopically injected into the mammary fat pad of female NSG mice. When tumors were palpable, mice were randomized and received a single i.v. injection of PBS as vehicle control, or MUC28z CAR T cells on day 4 post-tumor cell challenge. Tumor growth was monitored by caliper measurement. Data are presented as the mean ± SD. The statistical analysis was performed by two-way ANOVA. ***p < 0.001. **(B)** The wet weight of resected tumor mass on day 57 at endpoint. Top is the tumor pictures at the endpoint. The weights of individual tumors are presented. ***p < 0.001 (student *t*-test). **(C)** Increase of activation/exhaustion markers on CD8^+^ MUC28z CAR T cells. MUC28z CAR T cells were stained right before i.v. injection and right after tumor infiltrating lymphocytes analysis from tumor mass. Cells were gated on CD8^+^ T cells.

To assess the *in vivo* efficacy of MUC28z CAR T cells on TNBC tumor growth, we injected 5 × 10^6^ HCC70 tumor cells in the mammary fat pad of NSG female mice. Once tumors were established (4–6 days after tumor cell injection), a single i.v. injection of MUC28z CAR T cells (1 × 10^7^ cells) was administered to the HCC70 tumor-bearing mice. During the whole study period, no mouse death or significant illness was observed besides heavy tumor burden in the control group. Compared to the control, the growth of HCC70 tumors was dramatically reduced with a single injection of MUC28z CAR T cells at day 4 post-tumor cell inoculation, and this reduction was maintained until the experiment endpoint on day 57 (Figure 5A). The plot for individual mouse tumor measurement was included as Supplementary Figure 4A. The data for the wet weight of resected tumors showed changes that were consistent with the tumor growth data (Figure 5B). We also confirmed the tumor reduction by MUC28z CAR T cells in comparison to mock T cells. Again, a single injection of MUC28z CAR T cells at day 6 post-HCC70 tumor cell challenge was able to decrease tumor growth significantly (Supplementary Figure 4B).

We checked the MUC28z CAR T cells for CD25 and PD1 expression right before the adoptive transfer and on the day of the experimental endpoint. Data in Figure 5C showed the changes within CD8^+^ MUC28z CAR T cells. Approximately 50 days after surviving *in vivo*, the tumor-infiltrating CD8^+^ MUC28z CAR T cells expressed very high levels of CD25 as well as PD1 (Figure 5C), suggesting their further activation by *in vivo* tMUC1 tumor antigen stimulation.

### MUC28z CAR T Cells Retain Long-Term Efficacy for Decreasing HCC70 Tumor Burden *in vivo*

**Figure 6:**
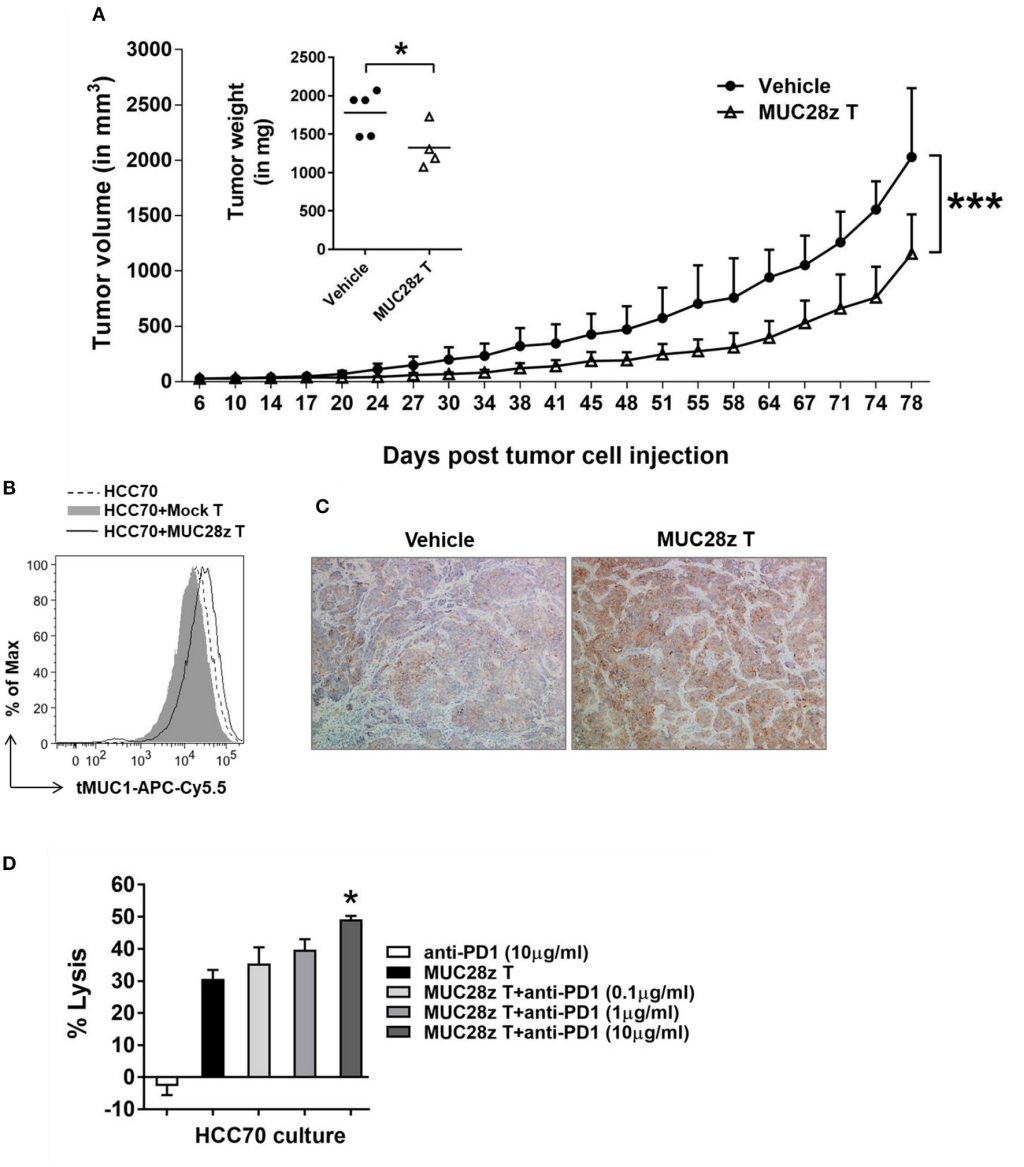
MUC28z CAR T cells have long-term efficacy for HCC70 tumor reduction *in vivo*. **(A)** Decrease of HCC70 tumor burden by a single injection of MUC28z CAR T cells *in vivo*. HCC70 tumor were inoculated as described in Figure 5. When tumors were palpable, mice were randomized and received a single i.v. injection of PBS as vehicle control, or MUC28z CAR T cells on day 6 post-tumor cell challenge. Tumor growth was monitored by caliper measurement. Data are presented as mean ± SD. The statistical analysis was performed by two-way ANOVA. ***p < 0.001. The insert shows the wet weight of resected tumor mass on day 81 at endpoint. *p < 0.05 (student *t*-test). **(B)** No tumor antigen loss while MUC28z CAR T cells were present *in vitro*. HCC70 cells were cultured alone or co-cultured with the mock T cells or MUC28z CAR T cells (E:T = 2:1) for 24 h. The viable HCC70 cells were analyzed for tMUC1 level. **(C)** Increased intensity of tMUC1 expression in MUC28z CAR T cells-treated HCC70 tumors. HCC70 tumor sections were prepared on day 81 at endpoint. Immunohistochemistry staining of tMUC1 was performed with TAB004 antibody. The brown staining shows tMUC1 positivity (100 × magnification). **(D)** Blocking PD1 enhanced the cytolytic response of MUC28z CAR T cells *in vitro*. HCC70 cells were co-cultured with MUC28z CAR T cells at E:T ratio of 2:1 for 24 h in the absence or presence of PD1 blocking antibody. The lysis of HCC70 cells was determined by MTT assay. Data are presented as the mean ± SEM. *p < 0.05 when compared to the group of MUC28z CAR T cells alone (student *t*-test).

To determine whether the anti-tumor effect of MUC28z CAR T cells could last longer than 57 days for controlling TNBC tumor growth, the HCC70 tumors were formed in NSG female mice same as described in Figure 5, followed by a single injection of MUC28z CAR T cells 6 days after tumor cell injection. Compared to the vehicle control, MUC28z CAR T cells effectively reduced the HCC70 tumor growth till the experiment endpoint on day 81 (Figure 6A). The plot for individual mouse tumor measurement was included as Supplementary Figure 5. The insert in Figure 6A showed the wet weights of tumors resected from NSG mice at the endpoint. The tumor weights in the MUC28z CAR T cell-treated group was significantly lower than the vehicle group that received PBS. However, it must be noted that even though there was a significant difference between control and treated groups, the tumors treated with MUC28z CAR T cells did start to progress faster after ~60 days post-treatment suggesting that (a) a single injection of CAR T cells may not be sufficient, (b) tMUC1 is lost in the remaining tumor that progressed, and (c) increased PD1 expression in the TILs (Figure 5C) blocks anti-tumor immune response and therefore a combination of CAR T cells with PD1 antibody needs to be assessed in the future.

We investigated tMUC1 expression in tumors post-MUC28z CAR T cell treatment *in vitro* and *in vivo*. The level of tMUC1 on HCC70 cells remained unchanged post co-culture with MUC28z CAR T cells or mock T cells *in vitro* (Figure 6B). In addition, the tumor sections from MUC28z CAR T cells and vehicle treated mice were stained with TAB004 for tMUC1. Surprisingly, there was increased tMUC1 staining in the group treated with MUC28z CAR T cells than in the vehicle group (Figure 6C) suggesting that tMUC1 loss is not a factor for tumor growth. Furthermore, the HCC70 cells cultured from the resected tumors showed similar levels of tMUC1 expression in both treatment groups (data not shown). Thus, tMUC1 tumor antigen down-regulation to avoid immune clearance by MUC28z CAR T cells is probably not involved. To assess if combining MUC28z CAR T cells with anti-PD1 antibody may be a better strategy, we pretreated MUC28z CAR T cells with PD1 blocking antibody at 0.1, 1, and 10 ug/ml concentration (clone EH12.2H7, Biolegend) and tested the lytic potential of MUC28z CAR T cells against HCC70 cells. The lysis potency of MUC28z CAR T cells was significantly enhanced with 10 ug/ml of PD1 antibody (Figure 6D). PD1 alone had no effect on the tumor cells.

## Discussion

Selecting the optimal tumor-associated antigen (TAA) as a target for CAR T cells is critical toward successful adoptive T cell therapy. The MUC1 protein is recognized as the second most targetable tumor antigen (29). MUC1 is expressed in over 90% of breast cancer, making MUC1 the most relevant and important antigen for breast cancer targeting (30). Furthermore, MUC1 is expressed on 95% of TNBC (19). However, due to the various challenges associated with adoptive immunotherapy and lack of antibodies that only recognize the tumor form but spares the normal form of MUC1 (8), there are only limited preclinical studies on MUC1-specific CAR T therapy. One is Dr. John Maher's group, one of the co-authors on this study who has been working on MUC1 CAR T cells using the HMFG antibody (8, 31) and recently, in the year 2016, Dr. Carl June's group published a new MUC1 CAR T cell using another MUC1(Tn) antibody (9, 32). Thus, there is a critical need for better tMUC1 targeting while sparing normal MUC1 for epithelial tumors. In the current study, the TAB004-derived MUC28z CAR T cells effectively reduced TNBC tumors both *in vitro* and *in vivo* (Figures 2, 5, 6). Since tMUC1 is widely expressed in most epithelial-derived solid tumors, including pancreatic ductal adenocarcinoma (26) and multiple subtypes of breast cancer (19), the MUC28z CAR T cells will likely have broad applications for solid tumor targeting.

In this study, we showed that the second-generation MUC28z CAR T cells derived from scFv of TAB004 (sequence published in patent US 20160130357 A1) significantly lysed TNBC tumor cells, and this activity strongly correlated with level of surface expression of tMUC1 (Figure 2). CD8^+^Myc-tag^+^MUC28z CAR T cells expressed high levels of CD25 and PD1. Despite their PD1 expression, MUC28z CAR T cells exhibited strong antigen-specific tumor lysis. This was in line with prior evidence that activated T cells and effector T cells in the early stages may express PD1 and remain functional (33, 34). Blocking PD1 signaling was able to enhance the tumor lysis by MUC28z CAR T cells *in vitro* (Figure 6D). Interestingly, some of the MUC28z CAR T cells also expressed CD11c. After the recognition of tMUC1, the levels of CD25 and CD11c were further upregulated (data not shown). CD11c^+^CD8^+^ T cells are a subset of activated, antigen-specific cytotoxic T cells that have strong antiviral effects *in vitro* and *in vivo* (28). The increase of CD11c expression on the CD8^+^MUC28z CAR T cells *in vitro* may indicate the expansion of tMUC1-specific cytotoxic T lymphocytes.

The antigen-specific production of Granzyme B, IFN-γ, IL-2, TNF-α, along with several other cytokines, further confirmed the activated and lytic phenotype of MUC28z CAR T cells (Figure 4). An IFN-γ neutralization assay demonstrated that IFN-γ significantly contributed to the MUC28z CAR T cell-mediated tumor killing albeit at low E:T ratios, which suggested that when there were fewer T cells around tumors, the contribution of IFN-γ might become dominant.

Most importantly, the HCC70 tumor reduction with a single injection of the MUC28z CAR T cells was very dramatic during the whole study course (Figure 5). This tumor reduction was maintained up to day 81 post-tumor challenge and mice had to be euthanized because tumor size in the control mice had reached the weight limit as set by the IACUC (Figure 6). As can be recognized from Figure 6C, tMUC1 expression on the tumor was retained at the end point of 81 days post-MUC28z CAR T cell treatment. This presence of tMUC1 after MUC28z CAR T cell treatment provided the rationale for repeated MUC28z CAR T cell administration to reach the maximal tumor reduction. It is of course plausible that tMUC1 expression is reduced in the early phases of MUC28z CAR T cell treatment *in vivo*; however, such *in vivo* kinetic experiments were beyond the scope of the present study. We did however assess the tMUC1 surface expression on HCC70 cells 6 and 24 h post co-culture with MUC28z CAR T cells. We observed no change in tMUC1 expression at either of the time points (data shown for 24 h; Figure 6B). Thus, it is unlikely that the escape mechanism for the tumor is the loss of tMUC1 antigen in this model. The increased CD25 and PD1 (Figure 5C) suggested that combination of MUC28z CAR T cells with anti-PD1 antibody might be beneficial as shown *in vitro* in Figure 6D. However, in a pilot experiment, anti-PD1 blocking antibody *in vivo* did not enhance the efficacy of MUC28z CAR T cells even though there was some efficacy of the combination *in vitro* (data not shown). Further, in a preliminary study, we detected an increased level of PD-L1 on HCC70 cells post co-culture with MUC28z CAR T cells *in vitro* (data not shown). Therefore, future experiments will investigate the combination of MUC28z CAR T cells with anti-PD-L1 antibody *in vitro* and *in vivo*. Some of the other escape mechanisms are down-regulation of surface tumor antigens (35), which we did not observe in our study (Figures 6B,C); functional impairment of T cells through immune checkpoints (36), and secretion of immunosuppressive cytokines by tumor and T regulatory cells (37, 38). We are currently exploring the role of cyclooxygenase 2 (COX-2) and indoleamine 2,3-dioxygenase (IDO) which are associated with tumor-induced immunosuppression. We found that TNBCs express high levels of the pro-inflammatory enzyme COX-2. The COX-2-specific inhibitor showed additive reduction of tumor cell growth in the presence of MUC28z CAR T cells *in vitro* (data not shown). The role of IDO inhibition in enhancing MUC28z CAR T cell tumor lysis is under investigation.

Together, the MUC28z CAR T cells reduced TNBC tumor growth effectively both *in vitro* and *in vivo*. Since a large variety of solid tumors of epithelial origin express tMUC1, we are currently exploring our MUC28z CAR T cell therapy for other tumor types, including pancreatic ductal adenocarcinoma. In addition, other potential combinatorial strategies are under investigation, which may lead to successful eradication of solid tumors.

## Author Contributions

RZ designed the study, conducted the experiments, acquired and analyzed data, interpreted the data, and wrote the manuscript. MY assisted in MUC28z CAR construct validation and data acquisition. LR assisted in TNBC cell culture and MUC1 detection. LW, AG, and JM provided the MUC28z CAR construct. JM also commented on the manuscript. PM provided intellectual input and reviewed the manuscript.

### Conflict of Interest Statement

PM is a board member of OncoTab, Inc. The remaining authors declare that the research was conducted in the absence of any commercial or financial relationships that could be construed as a potential conflict of interest.

## Abbreviations

TNBC: triple-negative breast cancer
MUC1: mucin 1
tMUC1: tumor form of MUC1
CAR: chimeric antigen receptor
PD1: programmed death-1
PBMC: peripheral blood mononuclear cell.
