# Corrigendum: CAR T Cells Targeting the Tumor MUC1 Glycoprotein Reduce Triple-Negative Breast Cancer Growth

**DOI:** 10.3389/fimmu.2020.628776

**Published:** 2020-12-07

**Authors:** Ru Zhou, Mahboubeh Yazdanifar, Lopamudra Das Roy, Lynsey M. Whilding, Artemis Gavrill, John Maher, Pinku Mukherjee

**Affiliations:** ^1^ Department of Biological Sciences, University of North Carolina at Charlotte, Charlotte, NC, United States; ^2^ School of Cancer and Pharmaceutical Sciences, King’s College London, Guy’s Hospital Campus, London, United Kingdom

**Keywords:** triple-negative breast cancer, immunotherapy, MUC28z CAR T cells, MUC1, TAB004

There was an error in the Funding statement. The correct name for the funder is DOD.

In the original article, we neglected to include the funder DOD, W81XWH-18-1-0711 to Ru Zhou, Mahboubeh Yazdanifar, Lopamudra Das Roy, Lynsey M. Whilding, Artemis Gavrill, John Maher, and Pinku Mukherjee.

The authors apologize for these errors and state that this does not change the scientific conclusions of the article in any way. The original article has been updated.

