# Supplementary material for: CAR T Cells Targeting the Tumor MUC1 Glycoprotein Reduce Triple-Negative Breast Cancer Growth
Source: Front Immunol. 2019 May 24;10:1149. doi: 10.3389/fimmu.2019.01149 (PMC6543840; doi:10.3389/fimmu.2019.01149)
Supplement: Supplementary file 1 [file Data_Sheet_1.docx]

**Supplementary Figure 1.**


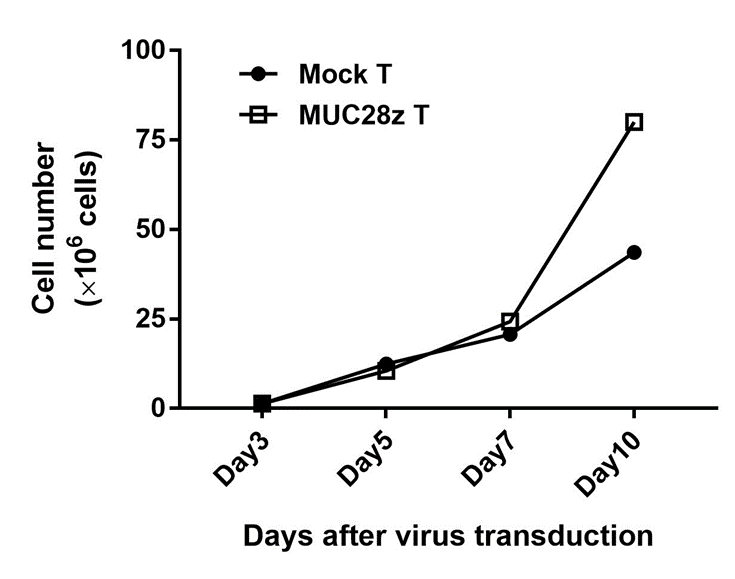


**Supplementary Figure 1. MUC28z T cells proliferate well *in vitro.*** Activated human PBMCs were transduced with MUC28z retrovirus or with medium only. Cells in culture were sampled and live cells were counted. Data shows one representative experiment out of three with similar trend.

**Supplementary Figure 2.**


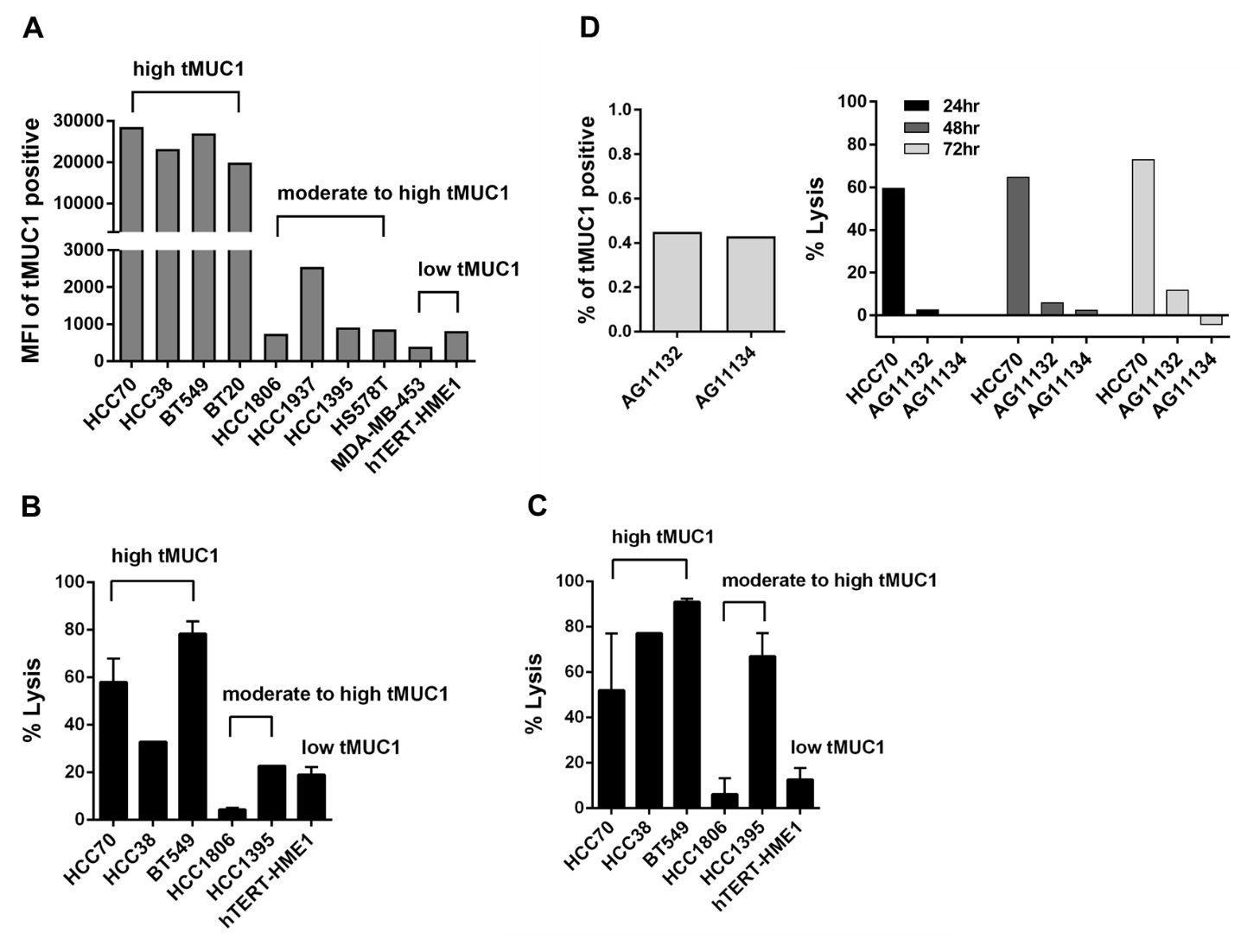


**Supplementary Figure 2. MUC28z CAR T cell mediate tMUC1-dependent TNBC tumor cell lysis *in vitro.*** (A) The mean fluorescence intensity (MFI) of tMUC1 positive cells from Figure 2A. (B and C) Percentage of TNBC tumor cell lysis by MUC28z CAR T cells. Cells were co-cultured at E:T ratio of 2:1 for 24h (B) or 48h (C). Tumor cell lysis was determined by MTT assay. Data are presented as the mean ± SEM. (D) Minimal cell lysis by MUC28z CAR T cells against two normal breast epithelial cells AG11132 and AG11134. Left panel: percentage of cells expressing tMUC1, determined by TAB004-APC/Cy5.5 staining and flow cytometry. Right panel: percentage of normal breast epithelial cell lysis by MUC28z CAR T cells. Cells were co-cultured at E:T ratio of 2:1 for 24h, 48h, or 72hr. HCC70 cells were used as positive control. Cell lysis was determined by MTT assay.

**Supplementary Figure 3.**


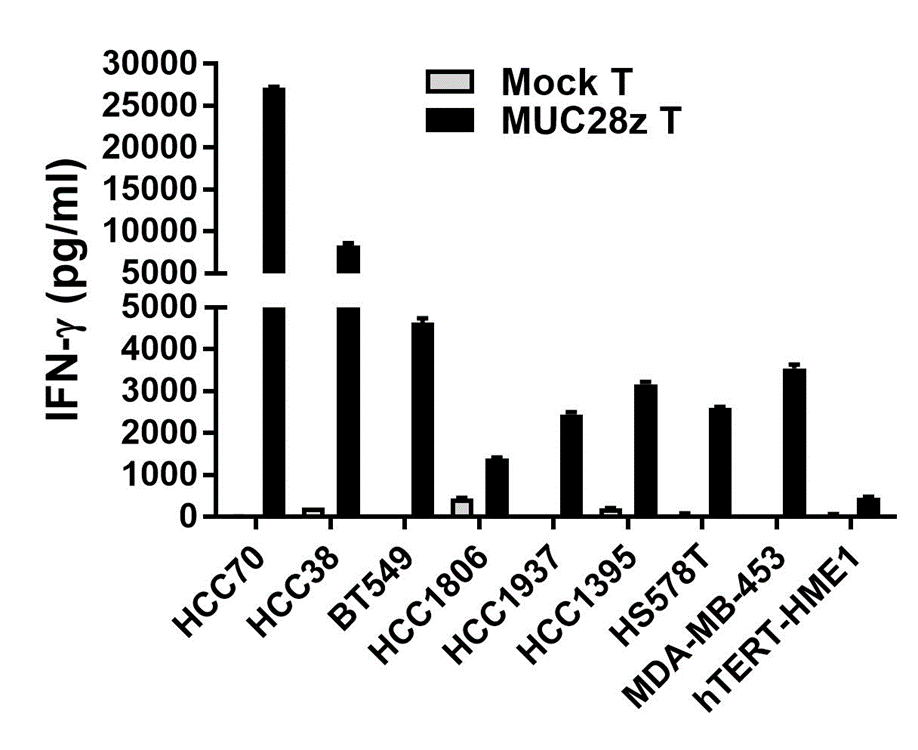


**Supplementary Figure 3. MUC28z CAR T cells secrete different amounts of IFN-γ** **when co-culture with TNBC cells.** T cells were co-cultured with the indicated tumor cell lines (E:T = 2:1) for 72hr, and then the culture supernatants were assayed for IFN-γ by ELISA. Data are presented as mean ± SD of replicates.

**Supplementary Figure 4.**


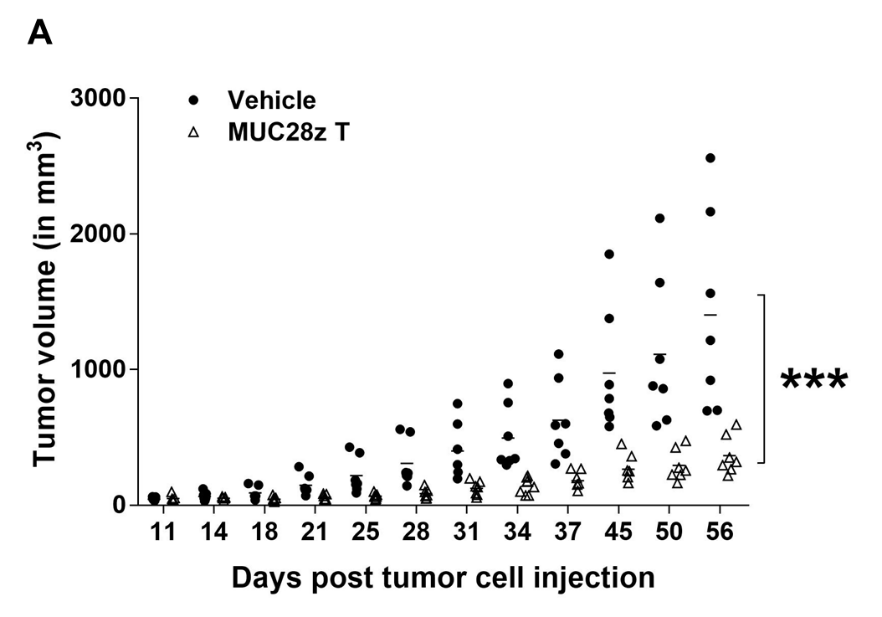


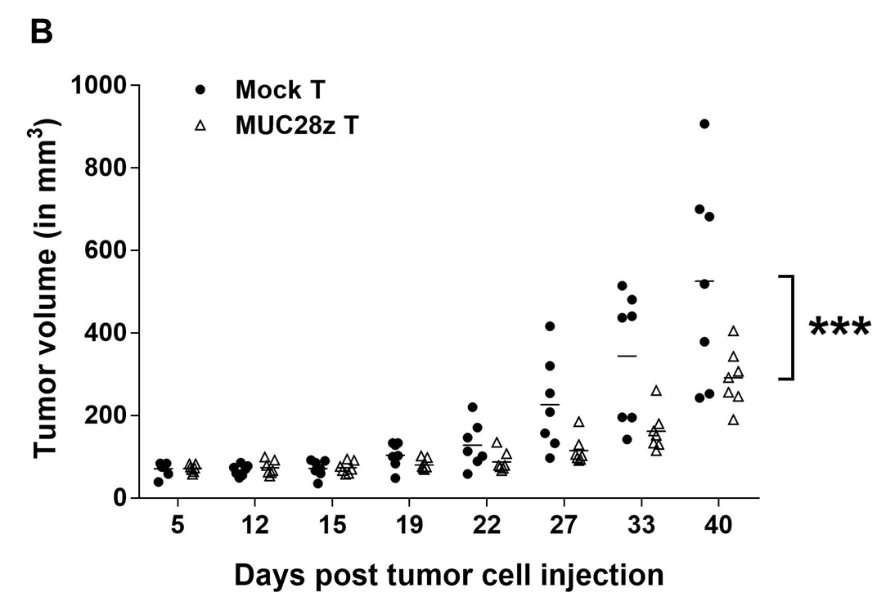


**Supplementary Figure 4. MUC28z CAR T cells reduce HCC70 tumor growth.** (A) The plot for individual mouse in tumor growth, as supplementary for summary data from Figure 5A. (B) HCC70 tumor growth reduction by a single injection of MUC28z CAR T cells *in vivo* when compared with mock T cells. HCC70 cells were orthotopically injected into the mammary fat pad of female NSG mice. When tumors were palpable, mice were randomized and received a single i.v. injection of mock T cells or MUC28z CAR T cells on day 6 post tumor cell challenge. Tumor growth was monitored by caliper measurement. Data are presented as the mean ± SD. The statistical analysis was performed by two-way ANOVA. ***, p<0.001.

**Supplementary Figure 5.**


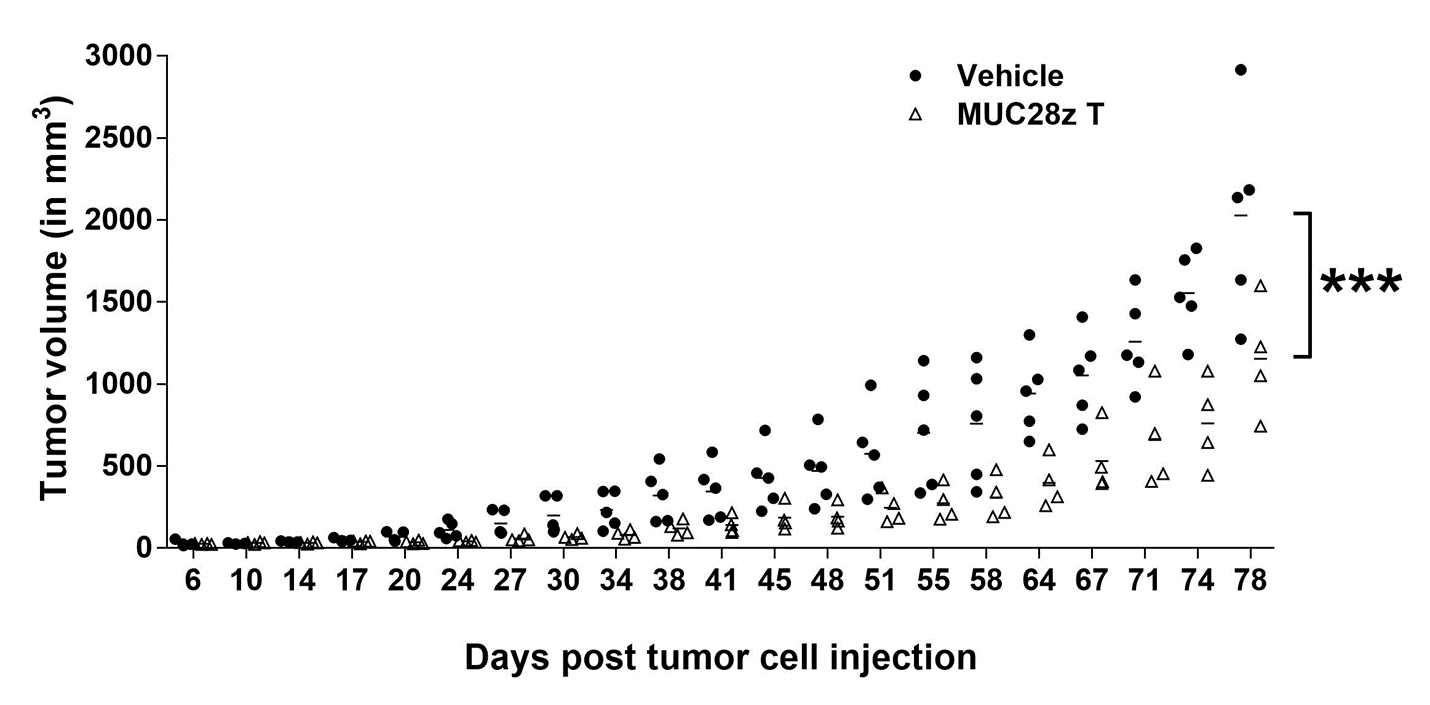


**Supplementary Figure 5. MUC28z CAR T cells retain long-term efficacy for decreasing HCC70 tumor burden *in vivo*.** This is the plot for individual mouse in tumor growth, as supplementary for summary data from Figure 6A.
